# Fan beam CT-guided online adaptive external radiotherapy of uterine cervical cancer: a dosimetric evaluation

**DOI:** 10.1186/s12885-023-11089-6

**Published:** 2023-06-26

**Authors:** Haibo Peng, Jie Zhang, Ningyue Xu, Yangang Zhou, Huigang Tan, Tao Ren

**Affiliations:** 1 Oncology Department, The First Affiliated Hospital of Chengdu Medical College, Chengdu, 610500 China; 2 Key Clinical Specialty of Sichuan Province (Oncology Department), The First Affiliated Hospital of Chengdu Medical College, Chengdu, 610500 China; 3 Clinical Medical School, Chengdu Medical College, Chengdu, 610500 China

**Keywords:** Online adaptive radiotherapy, Uterine cervical cancer, Image guided radiotherapy, In vivo dose verification, Dosimetry

## Abstract

**Purpose:**

To discuss the dosimetric advantages and reliability of the accurate delivery of online adaptive radiotherapy(online ART) for uterine cervical cancer(UCC).

**Methods and materials:**

Six UCC patients were enrolled in this study. 95% of the planning target volume (PTV) reached 100% of the prescription dose (50.4 Gy/28fractions/6weeks) was required. The patients were scanned with uRT-Linac 506c KV-FBCT then the target volume (TV) and organs at risk (OARs) were delineated by doctors. The dosimeters designed and obtained a routine plan (Plan0). KV-FBCT was used for image guidance before subsequent fractional treatment. The online ART was processed after registration, which acquired a virtual nonadaptive radiotherapy plan (VPlan) and an adaptive plan (APlan). VPlan was the direct calculation of Plan0 on the fractional image, while APlan required adaptive optimization and calculation. In vivo dose monitoring and three-dimensional dose reconstruction were required during the implementation of APlan.

**Results:**

The inter-fractional volumes of the bladder and rectum changed greatly among the treatments. These changes influenced the primary gross tumor volume (GTVp) and the position deviation of GTVp and PTV and positively affected the prescription dose coverage of TV. GTVp decreased gradually along with dose accumulation. The Dmax, D98, D95, D50, and D2 of APlan were superior to those of VPlan in target dose distribution. APlan had good conformal index, homogeneity index and target coverage. The rectum V40 and Dmax, bladder V40, the small bowel V40 and Dmax of APlan were better than that of VPlan. The APlan’s fractional mean γ passing rate was significantly higher than the international standard and the mean γ passing rate of all cases after the three-dimensional reconstruction was higher than 97.0%.

**Conclusion:**

Online ART in external radiotherapy of UCC significantly improved the dose distribution and can become an ideal technology to achieve individualized precise radiotherapy.

**Supplementary Information:**

The online version contains supplementary material available at 10.1186/s12885-023-11089-6.

## Introduction

Uterine cervical cancer (UCC) is the most common gynecological malignant tumor worldwide. Radiotherapy is applicable to all stages of UCC and is a radical treatment for locally advanced UCC and an auxiliary treatment for early- and middle -stage UCC with postoperative risk factors. With the application of intensity-modulated technology which represents precise radiotherapy, the radical rate of UCC has increased from approximately 40–70% in clinical practice and the toxicity and side effects have also been significantly reduced [1–3]. UCC radiotherapy includes external beam radiotherapy (EBRT), brachytherapy (BT) and their combination. EBRT plays an important role in the treatment of UCC, whose accuracy of dose delivery directly affects the final efficacy [4]. At present, multimodal image fusion improved accuracy of target area delineation, and image-guided technique significantly reduces systematic errors, such as set-up errors [5, 6]. However, due to the special anatomical position of the cervix, adjacent to the bladder and rectum with obvious changes in physiological status (varying due to the degree of filling), coupled with random factors such as tumor retraction, the target area may be deformed and moved between fractional treatments, which may lead to dose distribution deviation [7, 8]. Normally, the above impacts might be reduced by trying to complete positioning, planning and treatment under a relatively stable physiological state within half an hour because the anatomical morphology of the bladder and rectum does not change significantly [9–12].

Online adaptive radiotherapy (online ART) can modify plans based on real-time images and can be executed immediately to accommodate random transfer of target volume (TV) and organs, as well as the deformation of organs at risk (OARs) in the fractional treatments, to ensure the coverage of the target dose and to control the dose of OARs [13–15]. Online ART is particularly suitable for stereotactic radiotherapy of abdominal and pelvic tumors [16–21]. Online ART, offline ART and real-time ART are collectively known as adaptive radiotherapy (ART), of which the plan can be dynamically modified according to the anatomical and/or physiological changes of the patient, rather than maintaining a static plan throughout the treatment process, to ensure dose targeting [22–25]. Offline ART mainly targets systematic and progressive changes during treatment, such as patient weight loss and tumor morphologic changes. The new plan will be adopted in the follow-up fractional treatment. Offline ART has a significant effect on the clinical efficacy of head and neck and lung tumors [26–30]. Real-time ART can automatically modify and implement plans through continuous imaging, replanning and rapid dose calculation during treatment to reduce the impact caused by respiration, internal state changes and organ peristalsis or pulsation and achieve targeted tracking irradiation [31–34]. The concept of ART was put forward in 1990s and offline ART has obtained certain positive research results [26–30, 35, 36]. However, online ART and real-time ART are limited by technical conditions and other factors, so far their clinical applications are few.

This study conducted online ART for UCC based on daily CT images. We aimed to discuss the necessity of online ART for UCC by analyzing the volume changes of the bladder and rectum, the change rules of primary gross tumor volume (GTVp) and the planning target volume (PTV), and the relative deviation of the target geometric center before each treatment. By comparing the online adaptive plan with the virtual nonadaptive radiotherapy (V-non ART) plan, the dosimetric results and influencing factors of the online ART plan were analyzed, of which the dosimetric advantages were discussed to lay a foundation for adjusting the external boundary from the clinical target volume (CTV) to the PTV and the prescription dose segmentation method. The accuracy of dose delivery was confirmed by the statistical results of in vivo dose monitoring. Finally, our study will provide data support for the clinical promotion of online ART in the treatment of UCC.

## Materials and methods

### Clinical materials

Six uterine cervical cancer patients with EBRT indications in our department from March 2022 to May 2022 were included in this study. Three patients received postoperative radiotherapy and three patients received external radiotherapy before BT or surgery. Patients did not need para-aortic irradiation, did not have indwelling urinary catheter and can persist in lying flat for more than 30 min. The median age was 54.5 years old and the functional status score (Karnofsky, KPS) was greater than 90.

### Equipment

The uRT-linac 506c (United Imaging Healthcare Co., Ltd., Shanghai, China) is a CT-integrated linac guided by high-definition images, which was certified by China Food and Drug Administration(CFDA) as a radiotherapy device for treating systemic lesions in 2018. The uRT-linac 506c and radiotherapy plan management system (uRT-TPOIS, R001) integrate kilovoltage fan beam computed tomography (KV-FBCT) and a 6 MV X-ray linac, which are coaxial in the same bed, simultaneously realizing KV-FBCT image positioning and image guidance. The quality of the obtained image is consistent with the positioning image, which can be directly used for planning. The treatment planning system(TPS) has the functions of image fusion, automatic sketching, automatic planning, deformation and rigid registration and provides planning design methods, including 3-dimensional conformal radiotherapy (3DCRT), intensity-modulated radiotherapy (IMRT), volume-modulated arc therapy (VMAT) and stereotactic body radiation therapy (SBRT), which support online ART.

### Patient model and plan preparation

Patients were instructed to empty their bladders and rectums 2 h before CT localization and treatments and to drink 500 mL water 1 h in advance. KV-FBCT positioning scanning and image-guided scanning of uRT-linac 506c were adopted, with the abdominal helical scanning protocol. The scanning scope included the lower abdomen and pelvis, with a thickness of 5 mm. Seven beams (gantry angle: 180°, 130°, 80°, 30°, 330°, 280° and 230°; collimator angle: 0°; maximum dose rate 600 MU/min) of coplanar dIMRT were used. 95% PTV received 100% of the prescription dose (50.4 Gy/28 fractions/6 weeks) [37, 38]. The region of interest (ROI) template (naming and structural relationship processing of TV and OARs to be sketched), the optimized constraint template and the clinical goal sheet template were prepared (Table S1). The gross tumor volume of lymph node (GTVn) and the GTVp were treated with late-course incremental radiotherapy, which was not included in this study.

### Conventional radiotherapy plan formulation

KV-FBCT was used for localization scanning after effectively fixing the patient’s position, and the target area was then delineated according to the guidelines of the Radiation Therapy Oncology Group and the relevant international consensus [38–42]. GTVp refers to the primary lesion of cervical tumors. GTVn refers to pelvic metastatic lymph nodes. CTV generally includes vaginal stumps, common iliac, internal iliac, external iliac, obturator and presacral lymphatic drainage areas (non surgical cases also include GTV, cervix, uterine body, parauterine and partial vagina). PTV was formed by the expansion of CTV by 7–15 mm. The corresponding beam template and optimized constraint template were loaded for plan design and optimizing calculation. Finally, the conventional plan, named Plan0, was obtained. Plan0 was transplanted to the phantom for point and surface dose verification and it entered the treatment phase after passing the verification.

### Formulation of the online ART and V-non-ART plans

After the first reset of the patient, KV-FBCT image guidance was performed according to the preset scanning template. The positioning error was corrected under registration with the Plan0 image and then the online ART workflow was initiated, entering the patient’s intelligent planning module in the TPS and entering the online ART workflow after automatically loading Plan0. The OARs and couch in Plan0 were automatically delineated by deformation and target volumes (CTV and/or GTVp) in Plan0 were rigidly mapped to the current image and manually modified according to the Plan0 target area delineation principle. After confirming the ROI, an adaptive optimized calculation was selected based on Plan0 to generate the current adaptive radiotherapy plan APlanX (X = 1 ~ 28) according to the modified target area. The adaptive optimization algorithm used the dose distribution of Plan0 and the clinical goal sheet as inputs. The clinical goal sheet contained a wish-list with priorities, which was used to explicit the demands for prescription dose coverage of planning target and dosimetric criteria of OARs. The priorities were set to 1, 2 and 3, in which the number 1 represented the highest priority, and the number 3 represented the least priority (more details in supplementary material). Aplan was evaluated and then transmitted to treatment delivery application (TDA) for implementation once the dosimetric parameters of the ROI reached the pre-set dose objectives. In addition, the direct calculation was selected, and Plan0 was mapped to the current image, which further obtained the current virtual nonadaptive radiotherapy plan VPlanX (X = 1 ~ 28).

### Implementation of APlan and in vivo dose monitoring

An electronic portal imaging device (EPID) was used to monitor the in vivo doses of radiation fields. If the global γ passing rate was lower than 88% under the 3%/3mm benchmark, the current treatment was suspended or terminated, and the reasons were recorded and analyzed. The three-dimensional dose reconstruction was finally compared with the planned value.

### Statistical comparison of APlan- and VPlan-related parameters

The volume changes in the bladder and rectum were analyzed by comparing APlan and Plan0; the relative volume changes in the bladder, rectum, GTVp and PTV were calculated by the V (APlan-Plan0)/VPlan0 volume normalization method. [APlan offset value (x’, y’, z’) - Plan0 offset value (x, y, z)] and $\sqrt{{({\text{x}}^{{\prime }} - \text{x})}^{2}+{({\text{y}}^{{\prime }}-\text{y})}^{2}+{({\text{z}}^{{\prime }}-\text{z})}^{2}}$ were used to describe the deviation changes in the relative positions of the geometric center of the PTV and the GTVp. The dosimetric differences between the TV and OARs were observed by comparing APlan and VPlan.

### Statistical methods

SPSS 25.0 was used to perform statistical analysis on dosimetric parameters of TV and OARs, expressed by mean ± standard deviation. The Shapiro‒Wilk method was used to test the normality of the data. A paired sample *t* test was performed for data lines with a normal distribution, while the Wilcoxon signed rank test was performed with a significance level of 0.05.

## Results

### Volume changes of the bladder and rectum and volume change and position deviation of the PTV and the GTVp

**Fig. 1:**
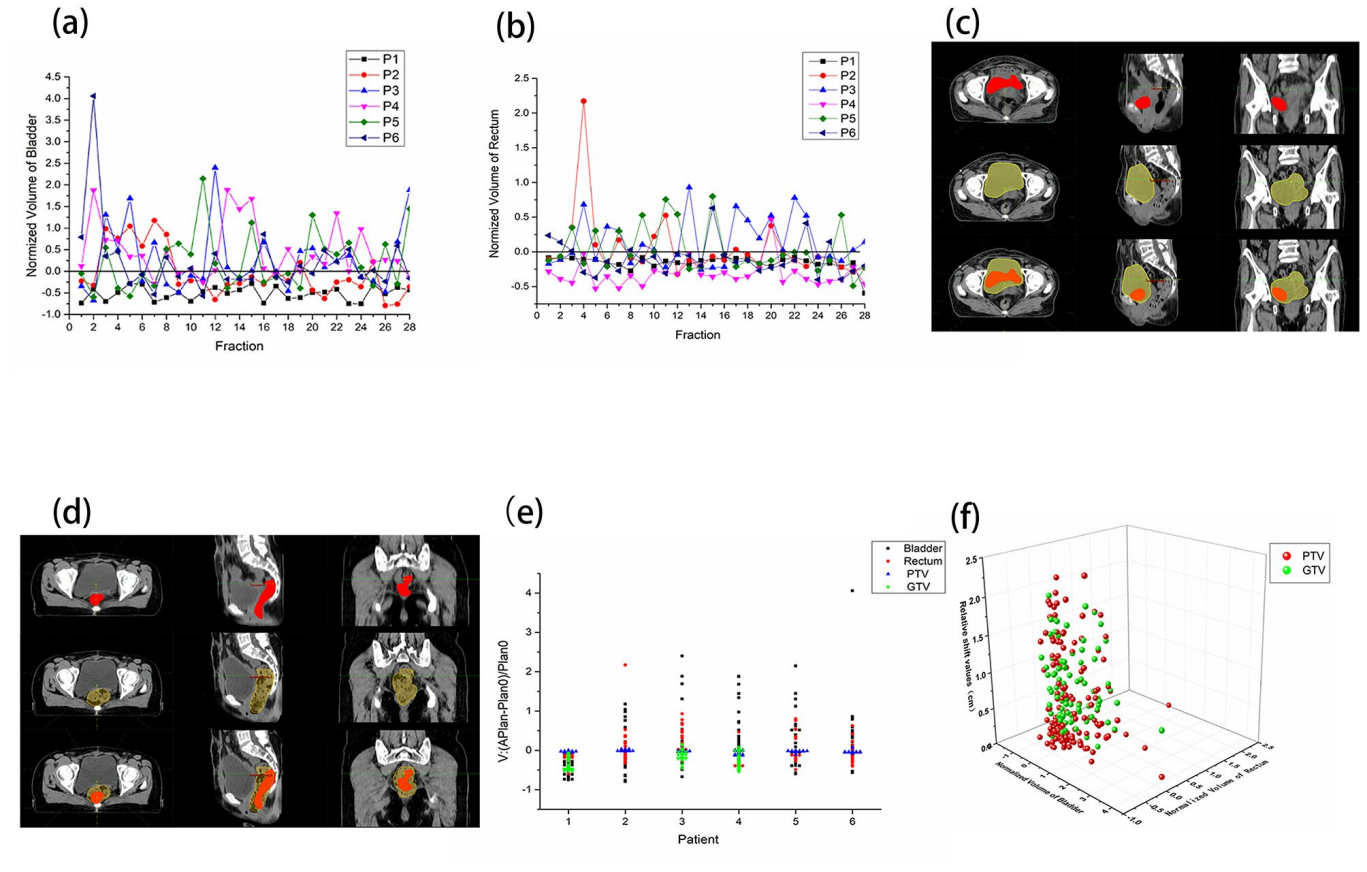
Relative changes in OARs, GTVp and PTV in patients. (**a**) Relative changes in bladder volume in each fraction. (**b**) Relative changes in rectum volume in each fraction. (**c**) Images of the initial and second treatments in case 6 (Top row: initial images. Middle row: daily images. Bottom row: merged images). (**d**) Images of the initial and fourth treatments in case 2 (Top row: initial images. Middle row: daily images. Bottom row: merged images). (**e**) Relative volume changes in the bladder, rectum, PTV and GTVp in each patient. (**f**) Effect of bladder and rectum volume changes on target volume deviation

As shown in Fig. 1(a) and Fig. 1(b), the volumes of the bladder and rectum in 6 cases changed significantly among fractional treatments, especially the bladder, but all cases were irregular. For example, the bladder volume in the second fraction of case 6 had the most obvious change with respect to Plan0, reaching 4.06, which is shown in Fig. 1(c), and the overfilled bladder was found in this division; the rectal volume of the fourth fraction of case 2 had the largest difference with respect to Plan0, which was approximately 2.17, as shown in Fig. 1(d), suggesting rectal inflation with the image analysis.

As shown in Fig. 1(e), the change of bladder volume was more obvious than that of the rectum, which was consistent with Fig. 1(a) and Fig. 1(b); the PTV and GTVp did not change significantly, but the GTVp decreased gradually with the accumulation of dose; the relative deviation changes of the geometric center point and “setup” point of the PTV and the GTVp in the lateral (LAT/X), vertical(VRT/Y) and longitudinal(LNG/Z) directions were obvious (the deviation ranges in the X, Y, and Z directions were -0.45~0.29 cm, -0.49~0.57 cm, -0.82~0.76 cm and -0.69~0.62 cm, -0.88~1.26 cm, -0.96~0.88 cm, respectively). As shown in Fig. 1(f), this deviation was affected by the volume changes of the bladder and rectum. The offset value was close to 0 when the volume changes of the bladder and rectum were small, and the points with large changes in the offset value had corresponding large changes in the bladder and rectum volume.

### Statistical results of dosimetric parameters

**Fig. 2:**
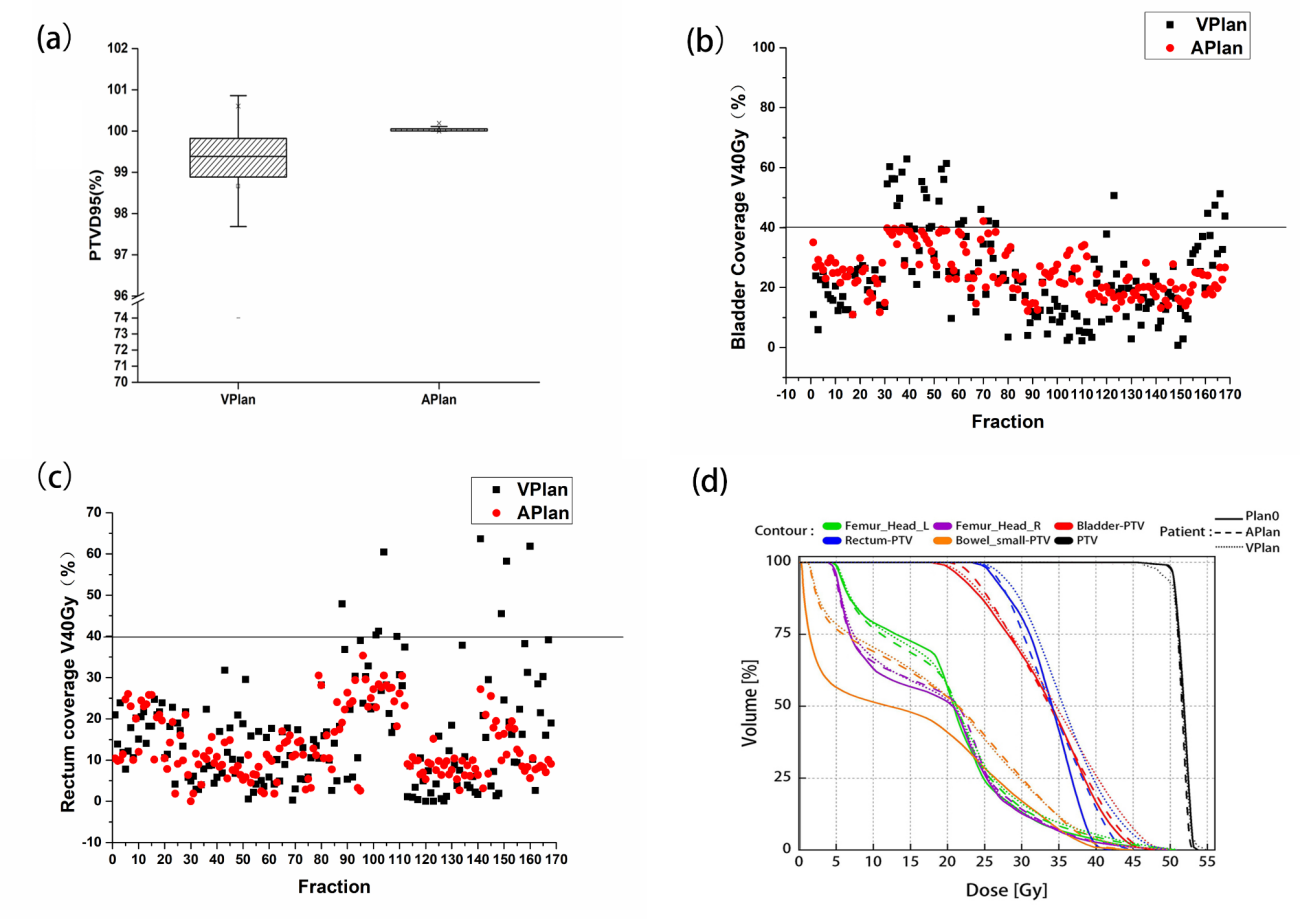
Dosage comparison between virtual planning (VPlan) and adaptive planning (APlan). (**a**) Doses of 95%PTV received in the two plans. (**b**) Bladder coverage with 40 Gy in the two plans. (**c**) Rectum coverage with 40 Gy in the two plans. (**d**) Dose volume histograms of one patient of the three plans

In terms of target dose distribution, as shown in Table 1, Dmax, D98, D95, D50 and D2 of APlan were superior to those of VPlan, with significant differences (p < 0.05), among which the D95 (5041.18 ± 42.02) of APlan met the prescription requirements, while D95 (4972.67 ± 159.17) of VPlan failed to meet the prescription requirements. Figure 2(a) also shows that the D95 of APlan reached 100% of the prescription dose, while most implementations of VPlan did not; APlan had good conformal index (CI = 0.92 ± 0.02), homogeneity index (HI = 0.05 ± 0.00) and target coverage (V100%=0.96 ± 0.01), while VPlan had significant differences with its corresponding parameters (0.82 ± 0.06, 0.10 ± 0.06, 0.90 ± 0.04, respectively) (p < 0.05).

**Table 1:** Comparison of dosimetric parameters of various planning target volumes

Parameter	Plan0	VPlan	APlan	VPlan V.S. APlan P values
Dmax(cGy)	5370.36 ± 12.65	5433.23 ± 35.48	5338.57 ± 18.66	<0.05
D98(cGy)	5010.42 ± 4.21	4835.04 ± 327.39	5005.33 ± 8.69	<0.05
D95(cGy)	5041.62 ± 1.75	4972.67 ± 159.17	5041.18 ± 42.02	<0.05
D50(cGy)	5182.73 ± 3.70	5178.26 ± 23.45	5155.27 ± 10.66	<0.05
D2(cGy)	5307.27 ± 2.98	5333.12 ± 28.48	5273.42 ± 13.33	<0.05
CI	0.90 ± 0.01	0.82 ± 0.06	0.92 ± 0.02	<0.05
HI	0.06 ± 0.00	0.10 ± 0.06	0.05 ± 0.00	<0.05
V100%	0.95 ± 0.00	0.90 ± 0.04	0.96 ± 0.01	<0.05

Abbreviations:Dmax: maximum dose; D98, D95, D50, D2: the dose received by 98%, 95%, 50%, 2% of PTV; CI: conformity index=(V_t,ref_/V_t_)·(V_t,ref_/V_ref_), V_t,ref_ is the target volume covered by the reference isodose line, V_t_ is the target volume, V_ref_ is the total volume covered by the reference isodose line; HI: homogeneity index=(D2-D98)/D50; V100%: coverage of 100% prescription dose on PTV.

The protection of OARs is shown in Table 2. The rectum V40 (13.42 ± 7.88) and bladder V40 (24.75 ± 7.63) of APlan were superior to the rectum V40 (15.91 ± 12.87) and bladder V40 (24.01 ± 14.66) of VPlan. The small bowel Dmax, V40 and rectum Dmax of APlan were better than those of VPlan (p < 0.05). Other parameters were equivalent. Figure 2(b) and Fig. 2(c) show the doses of bladder V40 and rectum V40 in 168 fractions of 6 patients, which suggested that the bladder V40 and rectum V40 of APlan were better than those of VPlan, but without statistical significance.

**Table 2:** Comparison of dosimetric parameters of various organs at risk

OAR	parameter	Plan0	VPlan	APlan	VPlan V.S. APlan p values
Bladder-PTV	V40(%)	22.41 ± 8.69	24.01 ± 14.66	24.75 ± 7.63	0.409
Rectum-PTV	Dmax(cGy)	4270.27 ± 181.21	4906.02 ± 316.91	4403.62 ± 85.33	<0.05
	V40(%)	2.74 ± 1.35	15.91 ± 12.87	13.42 ± 7.88	0.148
Small_ Bowel-PTV	Dmax(cGy)	4445.53 ± 173.81	4924.67 ± 235.47	4455.98 ± 40.73	<0.05
	V40(%)	0.65 ± 0.32	3.34 ± 3.27	2.50 ± 2.31	<0.05
Femur Head_L	V30(%)	10.89 ± 3.04	11.66 ± 3.75	11.82 ± 3.03	0.088
	V50(%)	0 ± 0	0.04 ± 0.16	0.02 ± 0.07	0.793
Femur Head_R	V30(%)	12.11 ± 1.55	12.16 ± 2.22	11.99 ± 2.16	0.174
	V50(%)	0 ± 0	0.01 ± 0.04	0.02 ± 0.12	0.372

Abbreviations:V30, V40, and V50: volume ratios of 30 Gy, 40 and 50 Gy covering the corresponding OAR.

The dose volume histogram (DVH) in Fig. 2(d) intuitively shows that the dosimetric characteristics of APlan are equivalent to those of Plan0 and superior to those of VPlan.

### Relative relationship between target dose and volume changes in the bladder and rectum

**Fig. 3:**
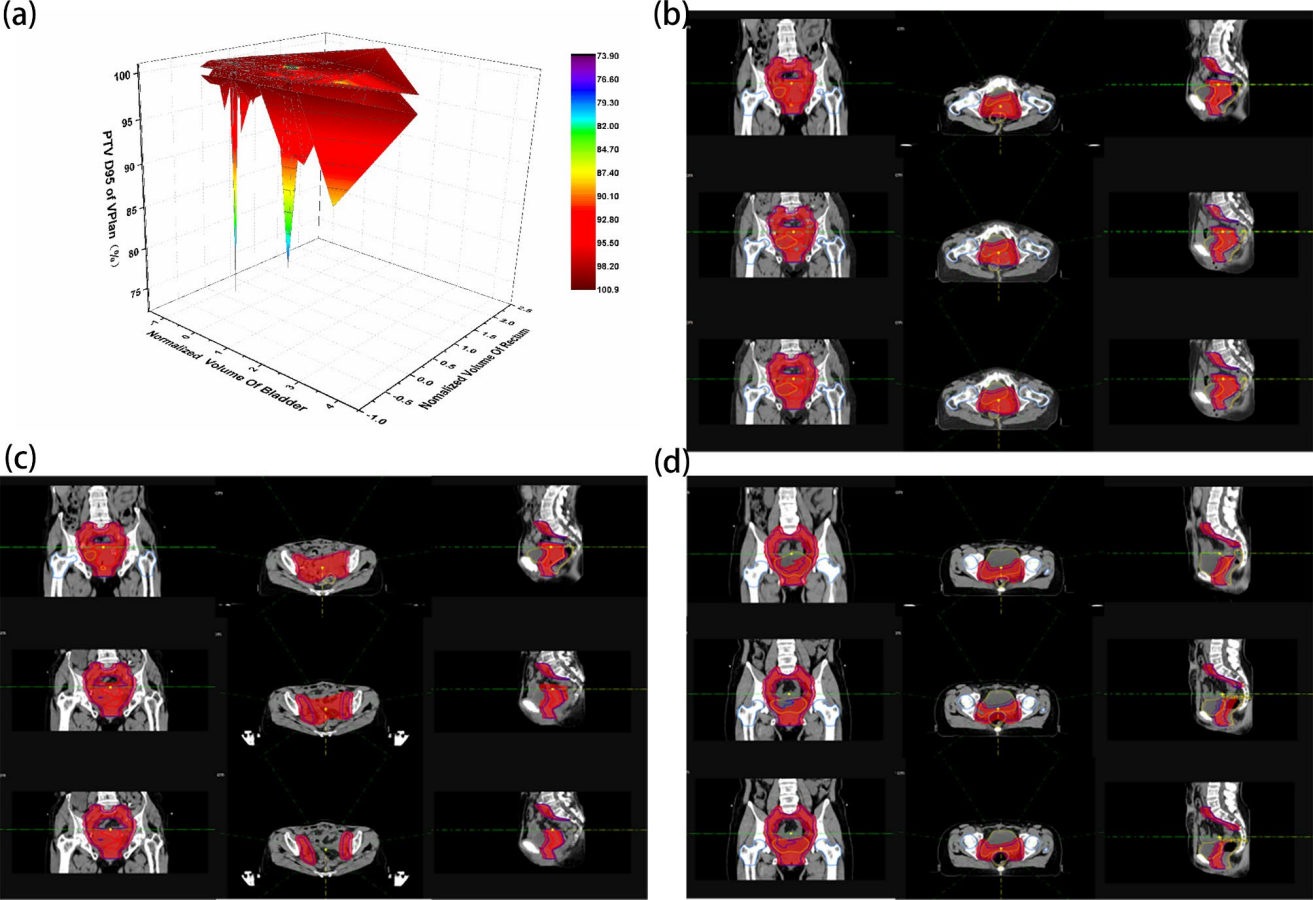
Effects of bladder and rectum volume changes on the dose distribution in the target volume. (**a**) The relationship between the volume changes of bladder and rectum and PTV D95 in VPlan. (**b**~**d**) Comparison of dose distribution in the three different plans. Plan0 in the top row, VPlan in the middle row, APlan in the bottom row, where (**b**) and (**c**) are the 7th and 28th fractions of a patient; the rectum volume of (**b**) decreased, and the GTVp of (**c**) decreased; (**d**) is the 20th fraction of a patient in whom the rectum was inflated and dilated

As shown in Fig. 3(a), the relative change in the VPlan target dose was positively related to the volume changes in the bladder and rectum, which directly affected the target dose distribution. Figure 3(b ~ d) shows the dose distribution in the target area under three different conditions (rectal volume reduction, GTVp reduction and rectal enlargement due to inflation). It was found that the target moved backward due to rectal volume reduction, and a low-dose area appeared in VPlan (Fig. 3(b)), while it was suggested in Fig. 3(c) that CTV (PTV) decreased due to GTVp shrinking and uterine body sinking, and the irradiated volumes of normal tissues and organs in VPlan were too large. Furthermore, Fig. 3(d) suggested that the rectal volume increased due to inflation and expansion, entering into the original treatment area in VPlan, with increasing irradiated dose and volume, which could be avoided by APlan.

### In vivo dose monitoring and three-dimensional dose reconstruction analysis

**Fig. 4:**
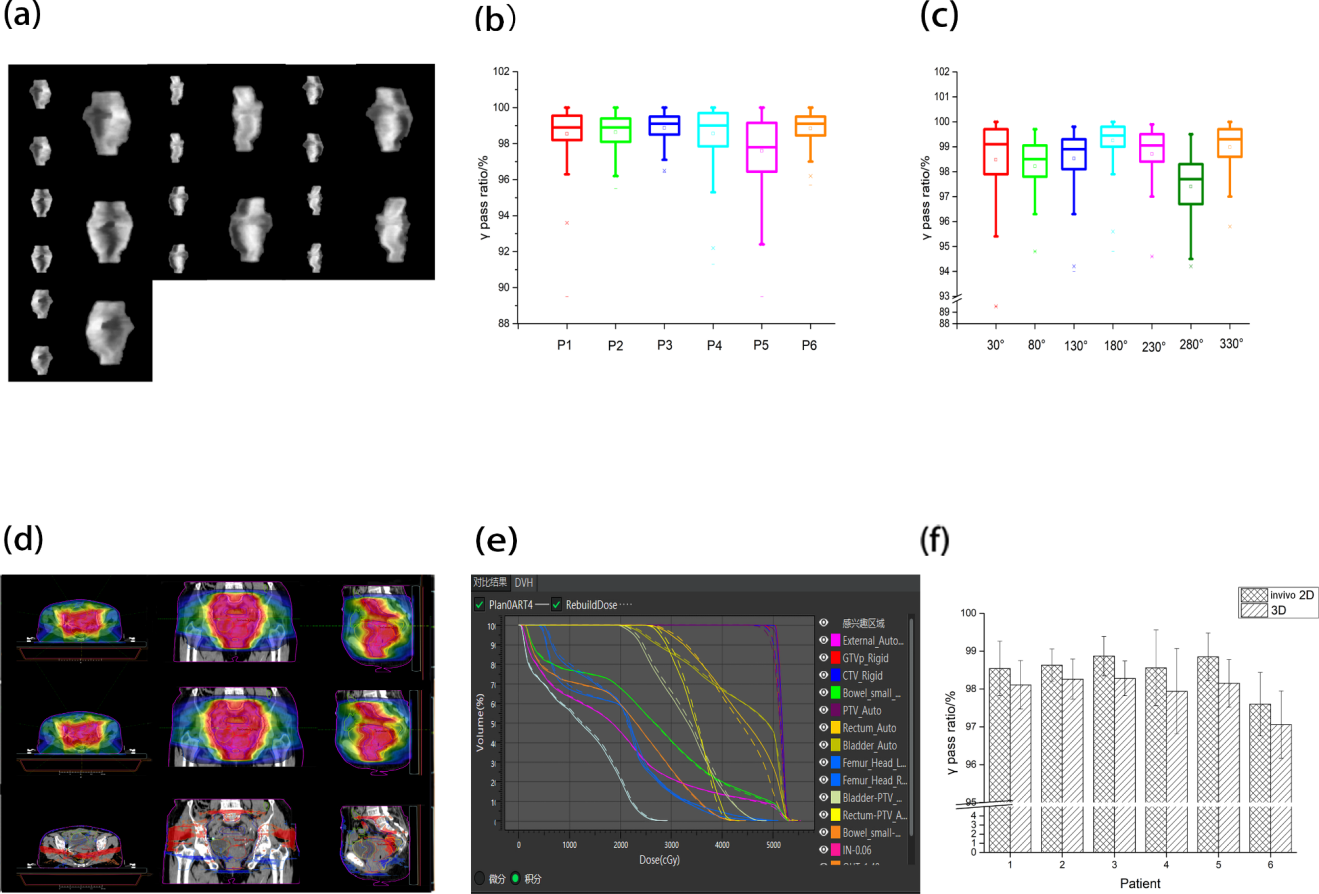
Dosage monitoring using in vivo method. (**a**~**c**) Results of two-dimensional in vivo dose monitoring. (**d**~**f**) Dose distribution results of the three-dimensional reconstruction

As shown in Fig. 4(a ~ c), the 168 fractions’ mean γ passing rate of APlan of 6 cases was significantly higher than the international standard [43, 44]. The mean passing rate of case 6 was slightly lower (97.6%) than others, of which the fractional passing rate was generally greater than 96.5%. The mean passing rates of the other cases were more than 98.5%, and their fractional passing rates were generally greater than 98.0%. The lowest passing rate was from case 4, though it was still close to 95.0%. The statistical results of all angles’ γ passing rate showed that the mean γ passing rate at 280° was approximately 97.5%, which was obviously lower than the other angles.

Three-dimensional reconstruction was carried out for the collected two-dimensional dose information, and the mean γ passing rates of each case were slightly lower than the corresponding two-dimensional mean γ passing rates, which were all higher than 97.0%. The dosimetric parameters of the TV and OARs in the three-dimensional reconstruction dose field were not significantly different from those of APlan (Fig. 4(d ~ f)).

## Discussion

Uterine cervical cancer (UCC) is a typical disease that is suitable and necessary for ART. It has been noted in UCC radiotherapy practice that tumor shrinkage, the movement and volume changes of peri-cervical OARs between fractions negatively affect the delivery of planned doses, which has the potential to reduce local tumor control and/or increase normal tissue toxicities [13, 45]. Based on IMRT and IGRT, performing the online ART may be the best means to achieve UCC clinical dosimetric goals. In this study, we found significant volume changes in the bladder and rectum relative to localized images through 168 fractional images of 6 cases and more significantly in the bladder, and all were irregular, consistent with other reports in the literature [7, 8, 46]. This may be related to factors such as altered human metabolism, surgery and radiation inflammation [47]. The volume changes of PTV generated by CTV expansion are not obvious, as the anatomy of the pelvis is solid and most of the lymphatic drainage areas in UCC are distributed along the pelvis wall [48]; approximately 80% of UCC cases are squamous cell carcinoma, which is relatively sensitive to radiation, which explains why the GTVp shrinks to different degrees with the accumulation of radiation dose – for example, by 60–80% in the first 3–4 weeks of radiotherapy [49–51]. This study also presented similar results. In addition, the volume changes of the bladder and rectum were found to affect the PTV and the GTVp, resulting in changes in the relative offset between the geometric center point and the “setup” point in the three-dimensional direction, and the GTVp was particularly significant in the vertical direction, reaching 2.14 cm. The points with large changes in offset values corresponded to larger changes in bladder and rectal volumes and vice versa.

Online ART has obvious dosimetric advantages in UCC radiotherapy and can deliver dose accurately. With the introduction of the ART concept, offline ART was gradually used in clinical research and practice, especially after the advent of kilovoltage cone beam computed tomography (KV-CBCT). The value of offline ART in adjusting the TV dose distribution and reducing the CTV-PTV border has been proved, and partly reduced the impact of tumor shrinkage to some extent. However, relative to the anatomical information of the thoracoabdominal and pelvic cavities of localization, offline ART cannot cope with the impact of random changes at each fractional treatment [52, 53]. To balance both of these effects, online ART was developed. Due to the technical characteristics and factors such as less fractions of BT for UCC, online ART had been successfully applied in the BT stage of UCC. In the last two decades, the technique has gradually matured and become routine [54–56]. Currently, EBRT for UCC is usually performed using coplanar IMRT or VMAT with conventional splitting of dose and 23–28 fractions, which is characterized by multiple fractions and long duration. In recent years, some scholars have randomized a few online ART studies based on CT guidance during the stage of EBRT for UCC, while there has not yet been reported on whole course online ART [57, 58]. EBRT was prescribed at a dose of 50.4 Gy for 28 fractions, five fractions per week, in all 6 cases of this study. Each time, the real-time images were reacquired, the TVs and OARs were redrawn based on the anatomical structure of the image feedback, and finally, the dose was optimized and the treatment was administered, completing the “localization–planning–treatment” regimen while the patient was almost stationary. In each case, 28 groups of APlan and VPlan and 1 group of Plan0 were obtained, reflecting the dosimetric differences between the two radiotherapy modalities. The dose distribution of Plan0 was directly mapped to the adaptive target area to form VPlan, and then the actual received dose of real-time TV and OARs was analyzed, which mainly showed that the target area coverage of the prescribed dose was insufficient, and the small bowel Dmax and rectum Dmax and V40 exceeded the standard or were even higher than the prescribed dose; the dose-optimized calculation was performed for real-time TV and OARs to obtain APlan. Its dosimetric parameters almost all met the standard, which was equivalent to Plan0, achieving a static effect with a dynamic adjustment.

Image quality and deformable alignment accuracy are key elements of Online ART, and device functionality is its foundation. The uRT-Linac 506c used in this study combines a diagnostic-grade KV-FBCT and a 6MV-X linac into a single device with two functions, image positioning and radiotherapy, and is equipped with integrated ART function software. KV-FBCT is guided by the same scan parameters as the positioning CT, and the acquired image sequence shares a CT value-relative electron density conversion curve with the positioning image so it can be used not only for pose calibration but also directly for planning design, which is the key to realizing online ART and is lacking in other forms of modal image guidance (e.g. CBCT, MR). In addition, the mechanical design of the CT and the linac are coaxial, with the same couch and the same coordinate system, and the couch board sinking monitoring and automatic correction function are added to keep the spatial position of the patient constant throughout the whole process. The Demons algorithm is used for variable image alignment, thus ensuring high image quality and high accuracy of alignment [59, 60].

EPID-based in vivo dose monitoring is a reliable guarantee of accurate APlan dose delivery. As the patient was placed on the couch during the whole process of online ART, pretreatment planned dose verification was not possible. Therefore, during the execution of APlan, in vivo dose monitoring was performed using EPID to analyze the γ passing rate in each field in real time and to set thresholds for warnings and interruptions according to the corresponding criteria [43, 44, 61–64]. The mean γ passing rate of 168 events in this study was significantly higher than the international standard, except for a slightly lower mean γ passing rate in the 280° incidence direction (possibly due to the variation of the equivalent thickness of the transmitted body), and the mean γ passing rates of the remaining angles were above 98.5%. The two-dimensional dose comparison was not sufficient to confirm its accuracy, so a three-dimensional reconstruction was also needed. The reconstructed dose distribution was analyzed by three-dimensional comparison with the planned presented dose distribution. The three-dimensional mean γ passing rates of all cases in this study were higher than 97.0%, indicating that there was no significant change in the anatomical environment of the target area during the process from APlan fractionated image acquisition to execution, and the APlan dose could be delivered accurately with the error controlled within 5% of the expected dose.

Online ART may change the clinical strategy in UCC. The shape and location of GTVp during online ART are largely determined, so simultaneous integrated boost of GTVp, during the EBRT stage, increasing the dose of the primary site, adjusting the dose splitting pattern to shorten the treatment time, all of which have the potential to improve the efficacy. The main driver of target area location shift comes from volume changes in the bladder and rectum. Even if the preparation before each radiotherapy treatment is consistent with that before positioning, the reproducibility of the shape of bladder and rectum cannot be improved or even vary significantly, with the largest relative changes in this study reaching 405.74% and 217.20%, respectively. Thus, online ART helps to omit the cumbersome preparation process, such as filling the bladder and emptying the rectum before positioning and treatment, thus improving patient comfort.

In this research, APlan presented dosimetric results with significant advantages and was accurately implemented. However, due to the small number of cases and the short follow-up period, it is not yet possible to confirm whether the final clinical benefit was achieved. There are still further detailed explorations are needed such as what are the trigger conditions of online ART, what are the principles of target volume adjustment, how to ensure the accuracy of dose accumulation in fading tumors and what are the possible risks of increasing the radiation dose of each KV-FBCT [65, 66]?, etc. All of these questions need to be further considered and studied. In addition, during the whole process of online ART, since patients are always immobilized on the couch, the time of the online ART workflow needs to be shortened as much as possible. Through this study, TV modification and APlan optimization calculations were time-consuming and the total time of online ART was 21 min on average. Therefore, more in-depth research is also needed in developing intelligent registration algorithms and automatic dose optimization algorithms to strive for further optimization and iteration [67].

## Conclusions

The application of online adaptive radiotherapy in UCC external radiotherapy can significantly improve the dosimetric distribution of radiotherapy for UCC; dose monitoring during treatment and 3D reconstruction of the dose after treatment can effectively guarantee the correct implementation of online adaptive radiotherapy. Further studies on image quality, deformation alignment and dose optimization algorithms can promote the application of online ART in UCC radiotherapy and may change some clinical strategies of UCC radiotherapy. Online ART has significant dosimetric advantages in UCC radiotherapy and is an ideal technique for achieving individualized and precise radiotherapy, with clinical promotional value.

## Electronic supplementary material

Below is the link to the electronic supplementary material.

Supplementary Material 1

## Data Availability

All data generated or analysed during this study are included in this published article [and its supplementary information files].

## Abbreviations

ART: Adaptive radiotherapy
BT: Brachytherapy
CBCT: Cone beam computed tomography
CTV: Clinical target volume
DVH: Dose volume histogram
EBRT: External beam radiotherapy
EPID: Electronic portal imaging device
FBCT: Fan beam computed tomography
GTVn: Gross tumor volume of lymph node
GTVp: Primary gross tumor volume
IMRT: Intensity-modulated radiotherapy
OAR: Organ at risk
PTV: Planning target volume
ROI: Region of interest
SBRT: Stereotactic body radiation therapy
TV: Target volume
UCC: Uterine cervical cancer
VMAT: Volume-modulated arc therapy
