# Supplementary material for: Fan beam CT-guided online adaptive external radiotherapy of uterine cervical cancer: a dosimetric evaluation
Source: BMC Cancer. 2023 Jun 26;23:588. doi: 10.1186/s12885-023-11089-6 (PMC10294475; doi:10.1186/s12885-023-11089-6)
Supplement: Supplementary file 1 — Supplementary Material 1 [file 12885_2023_11089_MOESM1_ESM.docx]

Supplementary material

**Details of adaptive optimization algorithm**

During optimization, the algorithm aimed to generate a very similar plan to the initial treatment plan which was mainly reflected in three aspects of 3D dose distribution including a similar dose falloff’s level for certain OARs, similar prescription dose conformity and DVH curve for planning targets. To achieve these goals, firstly, DVHs of the clinically concerned OARs were predicted by extracting the dose falloff features of these OARs on the initial plan. The optimization objective functions of OARs were then created based on the predicted DVHs. Secondly, the targets’ dosimetry parameters of the initial plan, including conformity index, dose at 2% and 98% volume, minimum and maximum dose were calculated, based on which the targets’ optimization objective functions of the adapted plan could be created. Considering the situation where the highest priority OAR presents a larger conflict with the planning target in adapted plan than the initial plan, meanwhile, the predicted OAR DVH fails to satisfy the highest priority goal, the algorithm will focus more attention to reducing this OAR dose than other OARs in order to satisfy this OAR’s clinical evaluation criteria. Furthermore, various optimization strategies, including OAR dose reducing method, dose conformity optimization and hot spot removal strategy, etc., are implemented in the algorithm to assure that the plan quality can satisfy the clinical requirements.

Table S1. Dose objectives and priorities of regions of interest

| **ROI** | **Clinical dose objective** | **Priority** |
| --- | --- | --- |
| PTV | Prescription dose=5040 cGy | 1 |
| PTV | maximum dose≤5392 cGy | 1 |
| PTV | 95% volume to receive≥100%  of prescription (5040 cGy) | 1 |
| Rectum-PTV | maximum dose≤4500 cGy | 1 |
| Rectum-PTV | ＜40% volume to receive≥4000 cGy | 1 |
| Femur_Head_R | ＜5% volume to receive≥5000 cGy | 3 |
| Femur_Head_R | ＜15% volume to receive≥3000 cGy | 3 |
| Femur_Head_L | ＜5% volume to receive≥5000 cGy | 3 |
| Femur_Head_L | ＜15% volume to receive≥3000 cGy | 3 |
| Bowel_small-PTV | maximum dose≤4500 cGy | 1 |
| Bowel_small-PTV | ＜40% volume to receive≥4000 cGy | 1 |
| Bladder-PTV | ＜40% volume to receive≥4000 cGy | 2 |
